# Influence des facteurs socio-économiques et du niveau d'éducation sur le cancer colorectal chez une population marocaine

**DOI:** 10.11604/pamj.2019.34.209.18345

**Published:** 2019-12-23

**Authors:** Fatima Ezzahra Imad, Houda Drissi, Nezha Tawfiq, Karima Bendahhou, Nadia Tahiri Jouti, Abdellatif Benider, Driss Radallah

**Affiliations:** 1 Institut des sciences du sport, Hassan I Université de Settat, Maroc; 2 Centre Mohamed VI Pour le Traitement des Cancers, Chu Ibn Rochd, Faculté de Médecine et de Pharmacie, Université Hassan II, Casablanca, Maroc; 3 Registre des Cancers de la Région du Grand Casablanca, Casablanca, Maroc; 4 Faculté de Médecine et de Pharmacie, Université Hassan II, Casablanca, Maroc

**Keywords:** Cancer colorectal, facteurs socio-économiques, niveau d’étude, stade de diagnostic, Colorectal cancer, socio-economic factors, education level, diagnosis stage

## Abstract

**Introduction:**

Le cancer colorectal demeure un véritable fléau et un problème majeur de santé publique. L'objectif principal de cette étude est d'identifier l'impact des facteurs socio-économiques et du niveau d'instruction sur la survenue du cancer colorectal et stade de diagnostic chez la population marocaine.

**Méthodes:**

Nous avons réalisé une étude cas témoin de janvier 2015 à janvier 2017 au Centre Mohammed VI pour le traitement des cancers. Deux cent vingt-cinq patients et 225 témoins éligibles et consentants sont interviewés de façon prospective en utilisant un questionnaire structuré et pré-testé.

**Résultats:**

L'âge moyen de nos patients était de 55,49 ± 14,06 ans. Cinquante-trois pourcent ont été diagnostiqués à un stade précoce du cancer du colorectal et 47% à un stade avancé. Par ailleurs, l'analyse détaillée de la population étudiée selon le statut socio-économique (SSE), montre que chez le groupe SSE faible, on note une proportion de 25,33% de patients contre 17,33% de témoins, tandis que dans le groupe SSE moyen, on retrouve des taux équivalents (45,33%) chez les patients et les témoins. Quant au groupe SSE élevé, le taux des patients ne représente que 16,89% contre 37,34% des témoins (P = 0,0001). Le niveau intellectuel et le niveau SSE étaient fortement corrélés au stade du diagnostic avec une différence significative. Ainsi 36,44% des analphabètes ont été diagnostiqués à un stade avancé contre 5,33 des patients ayant un niveau d'étude secondaire ou universitaire avec p = 0,02. De même 20,45% des patients de la catégorie SSE la plus basse étaient diagnostiqués à un stade tardif contre 5,33% des patients avec un SSE le plus élevé avec p=0,03.

**Conclusion:**

Nos résultats soulignent que le risque du cancer colorectal est fortement dépendant du niveau intellectuel et du statut socio-économique des patients. Une enquête plus approfondie est nécessaire pour clarifier les raisons de cette disparité.

## Abstract (en)

**Introduction:**

Colorectal cancer is a true scourge and a major public health problem. The main purpose of this study was to identify the impact of socio-economic factors and education level on the onset of colorectal cancer and of diagnosis stage in the Moroccan population.

**Methods:**

We conducted a case-control study of patients treated for cancer at the Mohammed VI center from January 2015 to January 2017. We interviewed, on a prospective basis and using a structured and pre-tested questionnaire, 225 patients and 225 eligible and consenting subjects.

**Results:**

The average age of patients was 55.49± 14.06 years. In 53% of cases diagnosis was made at early-stage colorectal cancer while in 47% at advanced stage. In addition, a detailed analysis of the studied population according to the socio-economic status (SES), showed a proportion of 25.33% (patients) versus 17.33% (control) in the low SES group, while, equivalent rates (45.33% patients versus 45.33% control) in the middle SES group. In the high SES group, the rate of patients was only 16.89% patients versus 37.34% control (p = 0.0001). Education and SES were strongly correlated with diagnosis stage, with a significant difference. Then 36.44% of illiterate patients were diagnosed in advanced stage versus 5.33% of patients who had completed their secondary education level or university course (p = 0.02). Similarly 20.45% of patients with low SES were diagnosed in late stage versus 5.33% of patients with higher SES (p = 0.03).

**Conclusion:**

Our results highlight that the risk of developing colorectal cancer is strongly dependent on the education and the socio-economic status of patients. A more thorough investigation is needed to clarify the causes of this inequality.

## Introduction

Avec plus de 1,4 millions de nouveaux cas diagnostiqués chaque année au niveau mondial [1], le cancer colorectal (CCR) est le troisième cancer le plus fréquent dans le monde: 275 000 nouveaux cas en Europe et 135 000 aux USA chaque année [2]. Le nombre de décès liés au CCR est évalué à 694 000 chaque année, ce qui en fait la quatrième cause de mortalité liée au cancer dans le monde [1]. Au Maroc, l'incidence standardisée du cancer colorectal est de 9,6 cas/1000000 habitants par an pour les deux sexes confondus [3]. De nombreux travaux scientifiques montrent que les facteurs socio-économiques ont un impact significatif à la fois sur le risque de cancer et son pronostic [4, 5]. A notre connaissance, il n'existe aucun travail portant sur les relations entre les facteurs socio-économiques et la prévalence des cancers colorectaux au Maroc. L'objectif principal de cette étude est de connaître le profil socio-économique des patients atteints de cancer colorectal admis au Centre Mohammed VI pour le traitement des cancers au Centre Hospitalier IBN ROCHD, comparés à des témoins indemnes de toute pathologie cancéreuse afin d'identifier d'éventuelles disparités sociales en matière de santé. Un objectif secondaire est de décrire de la façon la plus exhaustive et précise possible, en plus des données classiques sur les patients et la tumeur (âge, localisation tumorale, stade…), les variables pouvant être des facteurs socio-économiques pronostiques, qu'ils soient individuels (professions, études) ou géographiques (zone rurale ou urbaine).

## Méthodes

Il s'agit d'une étude cas-témoin menée au Centre Mohammed VI pour le traitement des cancers au Centre Hospitalier IBN ROCHD de Casablanca. Nous avons inclus dans notre étude de manière consécutive tous les patients atteints de cancers colorectaux confirmés histologiquement (n = 225), ayant été pris en charge au centre de janvier 2015 à janvier 2017. Le même nombre de témoins indemnes de toute maladie cancéreuse a été inclus parmi les patients admis au centre des consultations de dermatologie et d'ophtalmologie de CHU Ibn Rochd de Casablanca. Chaque témoin a été apparié à un patient sur le sexe et l'âge de ± 5 ans. Après accord du comité d'éthique local et consentement éclairé signé par le patient et le témoin après avoir reçu l'information nécessaire à sa prise de décision. Les données collectées dans le dossier médical ont été complétées par un questionnaire standardisé. Les variables du modèle de statut socio-économique ont été choisies de manière à tenir compte du contexte marocain et portaient sur: âge, niveau intellectuel, milieu de résidence, niveau socioéconomique, profession, assurance maladie, revenu mensuel, niveau d'instruction du conjoint, présence d'appareils électroménagers de base tel que (TV, radio, téléphone, machine à laver…), présence d'autres appareils complémentaires (climatiseur, ordinateur…), données cliniques et anatomopathologiques (localisation du cancer, histologie, classification TNM, stade et degré de différenciation).

Selon la classification TNM staging AJCC UICC 2017, les stades 1 et 2 ont été identifiés comme « stade précoce » et les stades 3 et 4 comme « stade avancé » [6]. En conséquence, nous avons calculé un score fait d'une somme de points donnés à chaque catégorie de la variable concernée. Puis, cette variable a été catégorisée en trois classes. En calculant le nombre de points, un score a été créé; plus le statut socio-économique (SSE) était élevé, plus le score l'était lui aussi, et inversement. Par exemple, la réponse d'un patient à une question sur le niveau d'instruction était codée comme suit: niveau universitaire-3 points; niveau secondaire-2 points; niveau primaire-1 point; Analphabètes-0 point; ne sait pas-0 point. Les réponses à la question “Avez-vous une TV/une radio/un climatiseur?” étaient notées comme suit: oui-1; non-0. Toutes les questions étaient codées de cette façon, ce qui a donné une échelle de 2 à 28 pour évaluer le facteur SSE. De ce fait, l'échantillon des patients a été réparti en trois classes, classes à SSE faible, à SSE moyen et à SSE élevé. Les patients qui ont obtenu moins de 10 points ont été classés à SSE faible, ceux qui ont obtenu de 11 à 20 points, sont catégorisés dans la classe à SSE moyen, et ceux qui ont eu 21 points ou plus, sont classés à SSE élevé. Nous nous sommes basés sur les paramètres de dispersion de la variable score: le 1^er^ quartile correspond à la 1^ère^ classe, le score se situant entre le 1er quartile et le 3^ème^ quartile correspond à la 2^ème^ classe, et au-delà du 3^ème^ quartile correspond à la 3^ème^ classe. Les analyses statistiques ont été réalisées avec le logiciel R. Le test de Chi^2^ a été utilisé pour la comparaison des groupes. Le seuil de significativité retenu est p < 0,05.

## Résultats

Parmi les patients interrogés au cours de la période 2015-2016, un total de 225 patients et 225 témoins a été inclus dans notre étude. L'âge moyen des patients était de 55,49±14,06 ans, avec des extrêmes allant de 18 à 90 ans, dont 119 hommes (52,9%) et 106 femmes (47,1%). Il y avait une prédominance masculine avec un sex-ratio de 1,12. La situation matrimoniale des patients est caractérisée par une prédominance des personnes mariées (77,78% des cas et 71,11% des témoins), suivie des célibataires (11,1% des cas 14,22% des témoins), des divorcées (9,33% des patients contre 9,78 des témoins) et des veuves (1,78% des patients contre 4,89 des témoins), sans différence significative. Le niveau d'étude montre de façon globale un niveau intellectuel bas chez les cas de cancer colorectal, comparés aux témoins avec une différence significative (p=0,0001). Ainsi 47,56% des patients sont des analphabètes contre 29,78% des témoins, 20% de patients ont un niveau primaire contre 16,89 des témoins et 16,89% des patients ont un niveau secondaire contre 24,44% des témoins. Le niveau universitaire ne représente que 5,78% des patients contre 22,22% des témoins. De plus, 32% des patients ont souligné qu'ils peuvent lire facilement un journal, contre 60% des témoins, avec une différence hautement significative (P = 0,0001).

Pour compléter la dimension culturelle, le niveau d'étude du conjoint est faible chez les patients par rapport aux témoins, avec une différence significative (P = 0.001). Ainsi, 61% des conjoints des patients ne peuvent déchiffrer un journal contre 48% des conjoints des témoins, avec une différence significative (P = 0.0001). La comparaison des appareils ménagers disponibles chez notre population d'étude permet de déduire que les patients sont nettement désavantagés, avec des différences significatives (P = 0,0001 pour l'ordinateur; P = 0,0001 pour le chauffe-eau et P = 0,0001 pour le climatiseur) (Tableau 1). La distribution des patients par catégorie socioprofessionnelle montre une prédominance des patients exerçant un travail physique par rapport à ceux exerçant un travail intellectuel, avec respectivement (73,77% des patients contre 58,22 des témoins; 26,23 des patients contre 41,78% des témoins). Cette différence était significative (p = 0,001). Quant aux régimes d'assurance maladie, leur répartition était significativement différente chez les patients et les témoins. Les patients affiliés au Régime d'Assistance Médicale (RAMED) étaient de 77,33% contre 32,89% des témoins (p = 0,0001). Sans conteste, le revenu mensuel est une des composantes essentielles de la mesure du SSE. Il est de façon globale bas chez les cas de cancer colorectal, comparé aux témoins avec une différence significative (p = 0,0001).

**Tableau 1:** Identification des 21 variables pour la construction de l’indice du SSE des patients et témoins

	Cas (%)	Témoin (%)
**Age moyen ± écart type**	**55 ,49 ± 14,06**	
**Statut matrimonial**	P = 0,89	
Célibataires	25(11,11)	32(14,22)
Mariés(es)	175(77,78)	160(71,11)
Veufs (ves)	4(1,78)	11(4,89)
Divorcé(es)	21(9,33)	22(9,78)
**Niveau d’étude**	P=0,0001	
Analphabètes	107(47,56)	67(29,78)
Primaire	67(29,78)	53(23,56)
Secondaire	38(16,89)	55(24,44)
Supérieur	13(5,78)	50(22,22)
Pouvez–vous lire le journal facilement		
Pas du tout	112(49,78)	61(27,11)
Avec difficulté	41(18,22)	29(12,89)
Facilement	72(32)	135(60)
	P=0,0001	
**Niveau d’étude de conjoint**		
Analphabètes	139(61,78)	107(47,56)
Primaire	36(16%)	38(16,89)
Secondaire	40(17,78)	59(26,22)
Supérieur	10(4,44)	21(9,33)
	P=0.008	
**Le conjoint peut lire le journal facilement**		
Pas du tout	138(61,33)	108(48)
Avec difficulté	42(18,67)	28(12,44)
Facilement	45(20)	89(39,55)
	P=0.0001	
**Tv**		
Oui	224(99,55)	224(99,55)
Non	1(0,45)	1(0,45)
	P=0,75	
**Ordinateur**		
Oui	92(40,89)	136(60,44)
Non	133(59,11)	89(39,56)
	P=0,0001	
**Réfrigérateur**		
Oui	210(93,3)	212(94,22)
Non	15(6,7)	13(5,78)
	P=0,42	
**Téléphone**		
Oui	208(92,44)	216(96)
Non	17(7,56)	9(4)
	P=0,07	
**Machine à laver automatique**		
Oui	161(71,55)	173(76,88)
Non	64(28,45)	52(23,12)
	P=0,1	
**Machine à laver semi-automatique**		
Oui	107(47,55)	113(50,20)
Non	118(52,45)	112(49,80)
	P=0,3	
**Chauffe-eau**		
Oui	100(44,44)	156(69,33)
Non	125(55,56)	69(30,67)
	P=0,0001	
**Climatiseur**		
Oui	11(4,89)	38(16,89)
Non	214(95,11)	187(83,11)
	P=0,0001	

Dans la population étudiée, 75,11% des patients résident dans un milieu urbain contre 83,11% des témoins et 24,89% des patients résident dans un milieu rural contre 15,5% des témoins, avec une différence significative (p = 0,038) (Tableau 1(suite)). La relation entre SSE et cancer colorectal a été étudiée dans notre échantillon. Nous avons trouvé que 25,33% des patients ont un SSE faible contre 17,33% des témoins, tandis que 53,33% des cas ont un SSE moyen contre 45,33% des témoins. Le SSE élevé ne représente que 16,89% des cas contre 37,34% des témoins. Cette différence dans le SSE des patients et témoins était significative avec p = 0,0001 (Tableau 2). L'analyse de nos résultats montre que les disparités SSE étaient plus élevées pour les cancers rectaux et ceux du côlon gauche que pour les cancers du côlon droit (Tableau 3). Nous avons constaté que le SSE bas était plus élevé chez les patients ayant un cancer de rectum (34,21%) que pour ceux ayant un cancer du côlon gauche (29,73%) ou un cancer du côlon droit (16,21% ), malgré que la différence n'est pas significative. Parallèlement, nous avons cherché l'association entre SSE des patients et le stade de diagnostic du cancer colorectal (Tableau 4). Selon la classification TNM, 53% ont été diagnostiqués à un stade précoce du cancer colorectal et 47% à un stade avancé. Concernant le niveau d'étude, 36,44% des analphabètes ont été diagnostiqués à un stade avancé contre 5,33% des patients ayant un niveau d'étude secondaire ou universitaire. Par ailleurs, plus les patients ont un niveau d'étude élevé plus le stade de diagnostic est précoce avec une différence significative p = 0,02. Le SSE était fortement associé au stade du diagnostic avec une différence significative. Les personnes de la catégorie SSE la plus basse (20,45%) étaient diagnostiquées à un stade tardif contre 5,33% des patients de la catégorie SSE la plus élevée. Au fur et à mesure que le niveau de SSE augmentait, les chances de retard au diagnostic diminuaient (Tableau 4).

**Tableau 1 (suite):** Identification des 21 variables pour la construction de l’indice du SSE des patients et témoins

	Cas (%)	Témoin (%)
**Assurance**	P=0,0001	
Pas d’assurance	7(3,11)	44(19,56)
RAMED	174(77,33)	74(32,89)
CNSS	34(15,11)	45(20)
CNOPS	6(2,67)	46(20,44)
Assurance privé	4(1,78)	16(7,11)
**Revenu mensuel**	P=0,0001	
Pas de revenu	42(18,67)	38(16,89)
<1000dh	10(4,44)	7(3,11)
Entre 1000-2000	22(9,78)	20(8,89)
Entre 2000-4000	102(45,33)	70(31,11)
Entre 4000-6000	39(17,33)	49(21,78)
Entre 6000-8000	14(6,22)	22(9,78)
>8000	1(0,44)	20(8,89)
**Qui paye pour vous les dépenses concernant votre pathologie**	P=0,0001	
Vous- même	99(44)	67(29,77)
Votre conjoint	16(7,11)	2(0,89)
Votre famille	110(48,89)	8(3,56)
**Pensez- vous que vous avez dépensé beaucoup d’argents pour votre maladie**	P=0,0001	
Oui, et ça m’a engendré des problèmes dans ma situation financière	185(82,22)	38(16,89)
Oui, mais ces dépenses restent gérables	26(11,56)	1(0,44)
Pas vraiment	9(4)	186(82,67)
**Milieu de résidence**		
Rural	56(24,89)	38(15,56)
Urbain	169(75,11)	187(83,11)
	P=0,038	
**Profession**	P = 0,001	
Travail physique (femme de foyer, agriculteurs)	166(73,77)	131(58,22)
Travail intellectuel	59(26,23)	94(41,78)

**Tableau 2:** Indice du SSE des patients et témoins

Statut socioéconomique		
	Cas	Témoin
Bas [0-10]	67(29,78)	39(17,33)
Moyen [10-20]	120(53,33)	102(45,33)
Elevé [20-30]	38(16,89)	84(37,34)
	P=0,0001	

**Tableau 3:** Statut socio-économique des patients en fonction du siège de la tumeur

	Niveau socio-économique		
Siège de la tumeur	Bas	Moyen	Elevé
Rectum	39 (34,21%)	58(50,88%)	17(14,91%)
Colon gauche	22(29,73%)	39(52,70%)	13(17,57%)
Colon droit	6(16,21%)	23(62,16%)	8(21,62)
P=0,3			

**Tableau 4:** Comparaison du stade de découverte en fonction de niveau d’étude et du statut socio-économique des patients

	Stade précoce (stade 1 et 2)	Stade avancé (stade 3 et 4)
**Niveau d’étude**		
Analphabètes	25(11,12)	89(36,44)
Ecole coranique; primaire	38(16,88)	29(12,88)
Secondaire; supérieur	39(17,33)	12(5,33)
	p=0,02	
**Statut socio-économique**		
Bas	21(9,33%)	46(20,45%)
Moyen	75(33,33%)	45(20%)
Elevé	26(11,56%)	12(5,33%)
	P=0,03	

## Discussion

A notre connaissance, il s'agit de la première étude réalisée dans le contexte marocain sur l'impact du statut socioéconomique et le niveau d'éducation, sur le risque de survenue du cancer colorectal et son stade de diagnostic. Nous avons démontré que le risque de cancer colorectal est plus élevé quand le niveau intellectuel est faible et le statut socio-économique est faible. Manser et Bauerfeind dans leur revue de la littérature ont noté une grande variation géographique concernant le lien entre le SSE et le risque de cancer colorectal. Aux États-Unis, un SSE faible est associé à une incidence accrue de CCR alors que l'association inverse est observée chez les Européens [6]. Nos résultats vont dans le même sens que les études américaines et à l'opposé des études européennes. Cette différence par rapport aux données des deux continents nous permet de soulever des hypothèses pour l'expliquer. Géographiquement notre pays fait partie du bassin méditerranéen réputé pour son régime riche en fruits et légumes qui permet de réduire le risque de cancer colorectal. L'augmentation du risque de CCR chez la population de faible SSE suggère que son alimentation est différente du régime méditerranéen et se rapprocherait plus de celle des USA avec fréquence de l'obésité, du tabagisme et la sédentarité. Par ailleurs malgré le dépistage du cancer colorectal pratiqué en Europe et aux USA, la différence en incidence du CCR réside dans l'accès au système de santé qui est plus facile en Europe par rapport aux états unis.

Dans notre contexte, hormis les différences d'accès aux soins liées au SSE, il n'existe pas à l'heure actuelle de dépistage organisé du CCR. Dans notre étude le SSE et le niveau intellectuel impactent aussi de façon significative le stade au diagnostic. Ainsi le cancer colorectal est diagnostiqué à un stade tardif quand le niveau intellectuel est bas et le statut socio-économique est faible. Cette association suggère une diminution des chances de survie et une augmentation du risque de mortalité par cancer colorectal chez cette population défavorisée. Ces données vont dans le même sens que toutes les études réalisées entre 1996-2010 qui rapportent une augmentation du taux de mortalité par CCR chez la population de faible SSE quelle que soit la région géographique où l'étude a été faite [7]. Le risque le plus élevé a été rapporté par Nitzkorski *et al*. avec un OR de 2,01 (95% CI, 1,66-2,43) [8]. Dans l'étude de Møller *et al*. qui a inclus 18159 patients le risque de décès était augmenté de 1.12/SES quintile [9]. Aarts *et al*. se sont concentrés sur l'association du SSE et du traitement du CRC dans leur étude. L'accès à la radiothérapie ou la chimiothérapie adjuvante était moins fréquent chez les personnes ayant un SSE faible (OR 0,4-0,99), ce qui indique une tendance continue des inégalités entre les groupes de SSE faible et élevé pour le traitement et les taux de survie et donc de la mortalité [10].

La corrélation du statut socio-économique et du siège de la tumeur a permis de démontrer la prévalence du cancer du rectum et du colon gauche par rapport au colon droit chez la population qui a le SSE le plus faible sans que la différence soit significative. L'absence de significativité de notre étude peut être expliquée par le faible nombre de patients participant à l'étude. Nos données vont dans le même sens que l'étude de DOUBENI qui a inclus 506488 participants et a démontré que les personnes ayant un faible SSE présentaient un risque plus élevé de développer un CCR cliniquement détectable, même après avoir pris en compte les autres facteurs de risque de CCR. Les disparités SSE étaient plus élevées pour les cancers du côlon gauche et rectum que pour les cancers du côlon droit [5]. Le dépistage est plus efficace pour le cancer du colon gauche et rectum par rapport au colon droit [11, 12]. Il a permis de réduire l'incidence et la mortalité par cancer colorectal. La participation au dépistage du CCR a été évaluée par Klabunde entre 2000 et 2008 dans la population américaine. Il a démontré des disparités dans les taux de dépistage du CCR au détriment des populations les plus défavorisées comparées aux groupes de haut SSE. Cette population est représentée par les hispaniques, de faible niveau d'instruction, de faible revenu et ayant des difficultés d'accès aux soins [13]. Ces données laissent penser que des actions visant à cibler la population au statut SSE défavorisée pour leur faciliter l'accès au dépistage peuvent réduire la différence de risque de CCR liée au SSE.

## Conclusion

Cette étude a confirmé l'hypothèse selon laquelle le SSE et le niveau intellectuel de la population étudiée avaient une influence sur la survenue et le stade de diagnostic du cancer colorectal. Cette influence semble plus importante pour le cancer du colon gauche et du rectum. Ces données pourraient être complétées par des études évaluant les habitudes alimentaires et l'IMC pour faire la part entre le mode de vie et la situation économique sur le risque du cancer colorectal. On peut à partir des données de notre étude proposer des actions visant à faciliter l'accès aux soins chez la population à faible SSE. Un programme de dépistage centré sur cette population pourrait réduire l'incidence du CCR et améliorer le diagnostic précoce pour augmenter les chances de survie dans cette population défavorisée.

### Etat des connaissances actuelles sur le sujet

- Le cancer colorectal représente un sérieux problème de santé publique dans le monde;
- Au Maroc, cette incidence est en constante croissance, il est devenu le premier cancer digestif, surclassant le cancer de l'estomac;
- La curabilité du cancer colorectal est en amélioration, grâce aux progrès thérapeutiques.

### Contribution de notre étude à la connaissance

- Ce travail est réalisé pour la première fois au Maroc, au Centre Mohammed VI pour le traitement des cancers;
- Cette étude a permis de connaître le profil socioéconomique des patients atteints de cancer colorectal admis au Centre Mohammed VI pour le traitement des cancers au Centre Hospitalier Ibn Rochd, comparés à des témoins;
- Notre étude a démontré que le niveau intellectuel et le statut socio-économique avaient une influence sur la survenue et le stade de diagnostic du cancer colorectal chez la population marocaine.

## Conflits d’intérêts

Les auteurs ne déclarent aucun conflit d'intérêts.
